# Psychological and social consequences of non-invasive prenatal testing (NIPT): a scoping review

**DOI:** 10.1186/s12884-019-2518-x

**Published:** 2019-10-28

**Authors:** Valérie Labonté, Dima Alsaid, Britta Lang, Joerg J. Meerpohl

**Affiliations:** 1 Institute for Evidence in Medicine, Medical Center – University of Freiburg, Faculty of Medicine, University of Freiburg, Breisacherstr. 153, 79110 Freiburg, Germany; 2 Clinical Trial Unit, Medical Center – University of Freiburg, Faculty of Medicine, University of Freiburg, Elsässer Str. 2, 79110 Freiburg, Germany

**Keywords:** Non-invasive prenatal testing, NIPT, Cell-free fetal DNA, cffDNA, Pregnancy, Down syndrome, Trisomy, Anxiety

## Abstract

**Background:**

Genomics-based noninvasive prenatal tests (NIPT) allow screening for chromosomal anomalies such as Down syndrome (trisomy 21). The technique uses cell-free fetal DNA (cffDNA) that circulates in the maternal blood and is detectable from 5 weeks of gestation onwards. Parents who choose to undergo this relatively new test (introduced in 2011) might be aware of its positive features (i.e. clinical safety and ease of use); however, they might be less aware of the required decisions and accompanying internal conflicts following a potential positive test result. To show the evidence on psychological and social consequences of the use of NIPT, we conducted a scoping review.

**Methods:**

We systematically searched four electronic databases (MEDLINE (Ovid), Cochrane Library (Wiley), CINAHL (EBSCO) and PsychINFO (EBSCO)) for studies that investigated the psychological or social consequences of the use of NIPT by pregnant women or expecting parents. The search was limited to studies published between 2011 and August 8, 2018. We identified 2488 studies and, after removal of duplicates, screened 2007 titles and abstracts, and then assessed 99 articles in full text (both screenings were done independently in duplicate). We included 7 studies in our analysis.

**Results:**

Five studies assessed anxiety, psychological distress and/or decisional regret among women with validated psychological tests like the Spielberger State Trait-Anxiety Inventory (STAI), the Pregnancy-Related Anxiety Questionnaire-Revised (PRAQ-R), the Kessler Psychological Distress Scale (K6) or the Decisional Regret Scale (DRS). Two studies assessed women’s experiences with and feelings after NIPT in interviews or focus groups. The included studies were heterogeneous in location, study setting, inclusion criteria, outcome assessment, and other characteristics.

**Conclusions:**

Only few studies on psychological consequences of NIPT have been identified. The studies assessed only short-term psychological consequences of NIPT at baseline and/or after receiving the results or after giving birth. Studies show that short term anxiety decreased when women received negative NIPT results and that decisional regret was generally low. We could not identify studies on long term consequences of NIPT, as well as studies on women’s partners’ short and long term outcomes, nor on social consequences of NIPT.

## Background

### Background of the study

Since the early 2010s, genomics-based noninvasive prenatal tests (NIPT) based on a blood sample from the pregnant women are offered in antenatal care to expecting parents who have a risk for certain chromosomal anomalies. The available NIPTs usually screen for all of the following seven chromosomal aneuploidies or for a subset of them: Down syndrome (trisomy 21), Edward syndrome (trisomy 18), Patau syndrome (trisomy 13), Turner syndrome (45,X), Klinefelter syndrome (47,XXY), Triple X syndrome (47,XXX) and Jacobs syndrome (47,XYY) [1].

The conditions caused by these aneuploidies vary considerably in severity and manifestations: Trisomy 21 is the most frequent aneuploidy in children that are born alive and causes mild to severe intellectual disability and several morphological features as well as possibly other medical conditions, such as heart disease. Some individuals can live independently as adults, others need substantial support and care [2]. Of children affected with trisomy 18 or 13 most die before birth or shortly after birth. The other syndromes that NIPT can screen for are aneuploidies of the sex chromosomes X or Y; generally, these have less severe health consequences for the persons affected. Examples in this background section will focus on trisomy 21, not only because it is the most frequent, but also because most expecting parents are familiar with it [3].

To analyze the fetal genome, NIPT uses cell-free fetal DNA (cffDNA) that circulates in the maternal blood and is detectable in maternal plasma from 5 weeks of gestation [1]. As NIPT is a screening test and not a diagnostic test, it only provides information about the possibility of having an affected fetus. The quality of tests is expressed as sensitivity (percentage of affected fetuses that are correctly identified) and specificity (percentage of non-affected fetuses that are correctly identified). In a meta-analysis, Badeau et al. reported a high accuracy of NIPT for the detection of trisomy 21, trisomy 18, and trisomy 13 in women at a high risk for fetal aneuploidy (sensitivities (95% confidence interval) from 95.8% (86.1 - 98.9%) to 99.7% (98.0 - 100%); specificities above 99%; almost all studies had a high risk of bias) [1].

Its high specificity for at least trisomy 21 sufficiently reassures many women who received a negative NIPT test result, so that there is no need for invasive diagnostic testing for confirmation of absence of these common chromosomal anomalies [4]. Women with a positive NIPT test result still do require an invasive test (e.g., karyotyping by chorionic villus sampling, CVS) for definitive confirmation because of the possibility of a false positive result. The likelihood of a false positive result for trisomy 21 for a 40 year old woman for example is 7.9% (given a prevalence of trisomy 21 of 1:85 [5]).

As there is no cure for chromosomal anomalies, continuation or termination of the pregnancy are the two options for expecting parents after discovery. An early detection of a chromosomal anomaly has certain advantages, such as more time for decision making, privacy (as fewer people are aware of the pregnancy), better management of pregnancy and birth, and safer methods of termination. On the other hand, 15% of trisomy 21 pregnancies are affected by spontaneous abortion at the end of the first or beginning of the second trimester (between 11 and 16 weeks), so that an affected fetus might naturally have been lost only shortly after discovery [4].

### Why this study should be made

NIPT is and has been seen as a breakthrough in prenatal screening for chromosomal anomalies because of its clinical safety and ease of use. Expecting parents who decide to undergo this relatively new test might only be aware of its positive features (i.e. no physical risk for the pregnant women or the baby) but they might be less aware of the possible upcoming decisions and internal conflicts affecting their pregnancy.

Green et al. conducted a systematic review (health technology assessment (HTA) report) on psychosocial aspects of genetic screening of pregnant women and newborns in 2004, before NIPT was introduced. They found that there was lack of evidence for a beneficial or reassuring effect of receiving a negative screening result; however, anxiety was raised in women receiving positive screening results. Up to 30% of women receiving a positive screening result in pregnancy expressed regret about their screening decision afterwards [6].

Bryant found, that undergoing NIPT as well as invasive testing, is often associated with high levels of anxiety (because of the fear of a positive result) [7]. Hodgson states that *“parents* [receiving a prenatal diagnosis of chromosomal anomaly] *frequently experience acute grief responses and strong emotions of guilt, anger, and loss”* [8]. As stress during pregnancy could possibly have negative consequences on the fetus (e.g. low birth weight) [9], clinicians, health policy makers, pregnant women, and their partners should be aware of the possibility of such consequences in advance.

Expecting parents or pregnant women in particular might perceive pressure to undergo NIPT, either from their social environment or from medical professionals. The decision against NIPT might even be seen as irresponsible, especially when women who deny NIPT, also deny invasive testing due to the risk of miscarriage [10].

Moreover, the marketing of the commercially available NIPT might simplify or even oversimplify its benefits. Advertisements and media coverage influence the perceived relevance of potential consumers who do not have an elevated risk for aneuploidies. Press articles on NIPT in the UK from a sample taken in 2014 showed a tendency to hype the benefits of NIPT [11].

Before undergoing such a test and coping with potentially negative results, psychological or social consequences of NIPT should be known. This aspect might become relevant on a public health scale and not only for subpopulations.

For informed choices on NIPT and possible consequences, obstetricians and gynecologists should be aware of the possible psychological and social consequences of NIPT for expecting parents and embed them in their deliberations. To our knowledge, no scoping or systematic review which investigates psychological and social consequences on NIPT has yet been published. While the diagnostic test accuracy and clinical utility of NIPT has been shown in systematic reviews (as for example in [1]), less is known on psychological and social consequences of its application. Therefore, we decided to conduct a scoping review on the psychological and social consequences of NIPT including a systematic search for studies assessing consequences of the use of NIPT.

## Methods

We chose a scoping review as a research format. This type of knowledge synthesis is suitable to identify existing evidence and research gaps in an emerging research area [12, 13]. In contrast to other evidence synthesis formats (as systematic reviews, for example), the quality of studies to be included in a scoping review is usually not assessed.

We based our method on the five steps of the original framework from Arksey and O’Malley [14] and a refinement by Levac et al. [15].

### Step 1: identifying the research question

We identified the research question in the context of a research project about the quality of the media coverage on NIPT in Germany: What is the evidence on psychological and social consequences of the use of NIPT?

### Step 2: identifying relevant studies

We developed a systematic literature search strategy (Additional file 1) with support of an information specialist (EM) [see Additional file 1]. We used a modified PICO-format to build our strategy around the aspects population (e.g. pregnant women, parents), intervention (e.g. NIPT, prenatal diagnosis/screening) and – to limit our search and thus receive a manageable number of results – outcome (e.g. maternal behavior, anxiety, social support). During testing of our search strategy, we omitted search terms that appeared to be relevant but did not add any relevant citations to the search result. Therefore, the search terms for different databases vary slightly.

We ran our searches on August 8, 2018, in the electronic databases MEDLINE (Ovid), Cochrane Library (Wiley), CINAHL (EBSCO) and PsychINFO (EBSCO). The searches were limited to studies published from 2011 onwards because NIPT was only introduced in 2011 [16]. Duplicates were removed using the algorithm of Bramer et al. [17].

### Step 3: study selection

We screened all unique references in two steps (title & abstracts, then full texts) by two reviewers (VL, DA) independently in the Covidence web application [18].

Inclusion criteria for both screening steps were:

- Population: pregnant women, parents
- Intervention: NIPT, cell-free fetal DNA (cffDNA)
- Outcome: psychological and social outcomes after NIPT

Study selection was not limited to a certain type of study, or by language.

Exclusion criteria: studies that did not match the above mentioned criteria. For example, studies on the decision-making processes or studies that were conducted before the introduction of NIPT.

### Step 4: charting the data

We extracted data of included studies in duplicate using a piloted, dedicated data abstraction form and summarized data narratively and in tables. Extracted data consisted of bibliographic information, study and women’s characteristics, outcomes relevant to our study question and additional data such as conflicts of interest.

### Step 5: collating, summarizing and reporting the results

General characteristics on the studies are shown in Table 1. We collated details and results of the included studies according to the types of outcomes that had been assessed in the studies (i.e. results from psychological tests (Table 2), women’s experiences (Table 3), and quotes from interviews or focus groups (Table 4). We also analyzed and summarized the results narratively.

**Table 1:** Characteristics of included studies

Study	Aim of study	Data collection	Participants	Outcome measures	Limitations of the study (excerpt), conflicts of interest (COI)
Farrell, et al., 2014, USA [19]	To determine how pregnant women conceptualize the utility of NIPT as compared to conventional screening and diagnostic tests.	Focus groups.	*N* = 53 women who received prenatal care at the study clinic; *n* = 10 women had NIPT. Mean age (range) = 31.7 ys (21–43) (*n* = 53); AMA^a^: *n* = 16.	Individual quotes.	Limited sample size, women from only one community. Authors declared to have no COI.
Lewis, et al. 2016, UK [20]	To report on a number of psychosocial outcomes including decisional uncertainty, distress and anxiety, as well as motivations for undergoing or declining NIPT and clinical service preferences.	Questionnaires at blood draw (Q1) and/or 1 month following receipt of results (Q2).	*N* = 582 women with a Down syndrome screening (DSS) risk > 1:1000 accepted NIPT free of charge as part of an implementation study in the UK NHS. *N* = 263 responded to Q1 and Q2 and were included in the analysis. Mean age (range): 35 ys (19–49) (*n* = 582). DSS^a^ risk distribution: > 1:1000/medium (*n* = 417); > 1:150/high (*n* = 165). NIPT results: Negative: *n* = 246; Positive: *n* = 10; Other: *n* = 7 (n = 4 test failed; n = 2 declined NIPT; n = 1 inconclusive results).	State-Trait Anxiety Inventory (STAI-6), short form^b^; Decisional Regret Scale (DRS)^c^.	Only a small number of women declined NIPT, no control group that has not been offered NIPT, low response rate to Q2, absence of baseline anxiety testing. Authors declared to have no COI.
Lo, et al., 2019, Hong Kong [21]	To assess decision outcomes (decision conflict, decision regret and anxiety) of pregnant women who are offered NIPT for high-risk Down syndrome screening results.	“first questionnaire” (Q1) and 4 weeks after Q1/after receiving NIPT results (Q2).	*N* = 262 women with positive Down syndrome screening results whose informed decision making about NIPT had been analyzed in a previous cohort study. Age distribution: < 35 years (*n* = 89); ≥35 years (*n* = 173). DSS^a^ risk distribution: 1:1–125 (*n* = 114); 1:126–250 (*n* = 91).	State-Trait Anxiety Inventory (STAI-6), short form^b^; Decisional Regret Scale (DRS)^c^.	No limitations discussed. Authors declared to have no COI.
Richmond, et al., 2017, Australia [22]	To examine the psychological impact of NIPT in women with a high-risk and low-risk result on combined first trimester screening (cFTS) and to examine factors influencing anxiety and decision-making in both risk populations.	Questionnaires at NIPT consultation and blood draw (point A) and 1 week post NIPT result (point B), then online after point B (point C).	*N* = 115 women requesting NIPT after combined first trimester screening (cFTS) but prior to morphology ultrasound were recruited from a genetic counsellor-led clinic (n = 2 women excluded due to failed NIPT). All *n* = 113 women received a negative NIPT result. Mean age (SD; range) = 36.4 ys (4.24; 27–44) (n = 113). cFTS risk distribution: ≤1:301/low (*n* = 50); ≥1:300/ high (*n* = 63). Both high-risk and low-risk cFTS groups had similar intrinsic trait anxiety levels at point A.	State-Trait Anxiety Inventory (STAI)^b^.	Failure to record reasons for non-participation and declining follow-up; bias against those that experience pathological anxiety; artificially inflated anxiety scores (completing STAI in a clinic environment); no control group without NIPT; cohort may demonstrate ascertainment bias towards those who both knew about and could afford NIPT; small study population. Authors declared to have no COI.
Takeda, et al., 2018, Japan [23]	To clarify the characteristics of psychological mental distress in postpartum women after non-invasive prenatal testing (NIPT) in Japan.	Questionnaires pre-NIPT and approx. 1 month post-partum.	N = 697 women that underwent NIPT at study hospital and had negative NIPT-results and low pre-NIPT psychological mental distress (K6) were included. Cases had high post-partum mental distress, controls had low post-partum mental distress (K6). Mean age (SD; range): ‘case’ group (*n* = 29): 37.9 ys (±2.4; 34–43); ‘control’ group (*n* = 668): 37.0 ys (±2.3; 30–41).	Kessler Psychological Distress Scale (K6), Japanese version^d^.	No control group with women who did not undergo NIPT, inability to adjust for the variable of neonatal abnormality. Authors declared to have no COI.
van Schendel, et al., 2017, Netherlands [24]	To address the questions whether women feel reassured and less anxious after receiving a favorable NIPT result and whether women feel satisfied with their choice for NIPT.	Questionnaires after NIPT counseling (Q1) and/or after NIPT or invasive test results were received (Q2).	N = 682 women participating in a study on evaluation of NIPT with an elevated first-trimester combined test (FCT) risk for aneuploidy (≥1:200) or based on medical history. Mean age (range): 35.8 years (22–45) (*n* = 682). Negative NIPT result: *n* = 656; positive NIPT result: *n* = 26. FCT^a^ risk distribution: ≥1:10 (*n* = 30); 1:11–1:100 (*n* = 267); 1:101–1:200 (n = 267); missing (*n* = 24); NA (*n* = 92).	Spielberger State-Trait Anxiety Inventory (STAI-6), short form; Dutch version^b^; Pregnancy-Related Anxiety Questionnaire-Revised (PRAQ-R scale)^e^; Survey on Reassurance/ Satisfaction/ Experience with NIPT.	Low response rate to post-test questionnaire, inability to perform subgroup analyses for positive NIPT results group, possible selection bias due to several reasons. COI: One middle author had been employed and one middle author had participated in clinical research sponsored by companies that offer NIPT
Vanstone, et al., 2015, Candada [25]	To examine how Ontario women have experienced the process of publicly funded NIPT in 2014, with the aim of identifying women’s values about this process to inform future formal policy making about this new health technology.	Interviews and constructivist grounded theory.	*N* = 38 women at ‘high risk’ of fetal aneuploidy, identified at prenatal diagnostics unit, via advertisements, snowball sampling and personal networks until theoretical saturation was reached. Mean age at delivery = 35.4 ys (*n* = 38). Age classes: 25–29 ys (n = 2); 30–34 ys (*n* = 14); 34–39 ys (*n* = 16); ≥40 ys (n = 6).	Grounded theory and individual quotes.	Particular group of women, older and more educated than the average, with potentially more thorough understanding of NIPT and the available testing options. Authors declared to have no COI.

^a^*AMA* advanced maternal age (≥35 years at delivery), *cFTS* combined first trimester screening, *DSS* down syndrome screening, *FCT* first-trimester combined test, *NIPT* non-invasive prenatal testing, *SD* standard deviation

^b^Spielberger State-Trait Anxiety Inventory (STAI): self-evaluation questionnaire to differentiate between “the temporary condition of ‘state anxiety’ and the more intrinsic quality of ‘trait anxiety’” [22], 2 × 20 items, range 20–80 (Richmond 2017: 20–90), higher scores indicate higher anxiety. Scores ≥50 indicate elevated state anxiety. Score ≥ 40 on the STAI trait scale are considered ‘highly anxious’. Van Schendel considered a score of 34–36 as normal anxiety. Lewis 2016, Lo 2019, van Schendel 2017 used a short version with 6 items (STAI-6) and considered a cut-off 31–49 as average anxiety

^c^Decisional Regret Scale (DRS): measure of distress or remorse after a health care decision, range 0–100, higher scores indicate a higher level of regret, no formal cut-off, a score of ≥50 indicated decisional regret in Lo 2019

^d^Kessler Psychological Distress Scale (K6): assesses frequency of experienced symptoms during the past 30 days (six items), range: 0–24, K6 score ≥ 10 is defined as a high score in Takeda 2018

^e^Pregnancy-Related Anxiety Questionnaire-Revised (PRAQ-R) Scale: Child-related anxiety measured by subscale ‘fear of bearing a handicapped child’ (four items), range 4–20, higher scores mean higher levels of child-related anxiety

**Table 2:** Data from studies assessing outcomes with validated psychological tests like STAI, DRS, K6

Study	Test	Data source	Baseline (at NIPT blood draw or counselling)	After receiving tests results	Results
Lewis, et al. 2016, UK [20]	State anxiety (STAI-6, short form, range 20–80)	*N* = 263 women with either negative or positive NIPT results; questionnaires at baseline (Q1), and 1 month following receipt of results (Q2).	STAI-6 mean score (SD) = 40.1 (±15.5).	STAI-6 mean score (SD) = 34.3 (±12.6).	Decrease of anxiety.
Richmond, et al., 2017, Australia [22]	State anxiety (STAI, range 20–90)	N = 113 women at baseline (point A); *n* = 83 women at least 1 week after receipt of results (point C, online); all with negative NIPT result.	STAI mean score (SD) in high-risk cFTS vs. low-risk cFTS = 42 (±11) vs. 36 (±11); *p* < 0.01.	STAI mean score (SD) in high-risk cFTS vs low-risk cFTS = 30 (±11) vs. 29 (±8); *p* = 0.61.	Whilst the high-risk cFTS population had significantly higher levels of state anxiety when they elected NIPT, both groups experienced a statistically significant reduction in state anxiety to similar final levels after they received a negative NIPT result (p < 0.01).
van Schendel, et al., 2017, Netherlands [24]	State anxiety (STAI-6, range 20–80)	*N* = 656 women with negative NIPT result.	STAI-6 mean score = 44.3.	STAI-6 mean score = 28.8.	Significant reduction in state anxiety in women with negative NIPT result (*p* < 0.001).
Lewis, et al. 2016, UK [20]	Elevated anxiety (STAI-6, short form, scores ≥50)	N = 263 women with either negative or positive NIPT results; questionnaires at baseline (Q1), and 1 month following receipt of results (Q2).	Rate of women: 29.9% (*n* = 174).	Rate of women: 13.7% (*n* = 36).	Significant decrease in [elevated] anxiety at time of Q2. Of the 36 women whose scores indicated elevated anxiety, 30 had a negative NIPT result, 5 had a positive NIPT result (confirmed through invasive testing) and 1 had an inconclusive result (the fetus was found to be unaffected following invasive testing).
Lo, et al., 2019, Hong Kong [21]	Elevated anxiety (STAI-6, scores ≥50)	N = 254 women at baseline (Q1); *n* = 229 women 4 weeks later and after receiving results (Q2).	STAI-6 score ≥ 50: *n* = 142/254 women (55.9%).	STAI-6 score ≥ 50: *n* = 59/229 women (25.8%).	Elevated anxiety was less common after the results had been announced (p < 0.001). Elevated anxiety was not more common among NIPT decliners than acceptors (*p* = 0.679).
van Schendel, et al., 2017, Netherlands [24]	Elevated anxiety (STAI-6, score ≥ 50)	N = 26 women with positive NIPT results.	(−-)	STAI-6 mean score = 54.0.	Overall, the 26 women who had received a positive NIPT result for trisomies 21, 18 or 13, or for other trisomies showed high anxiety scores after receiving test-results (M = 54.0). For 11 of 14 women who had had confirmatory invasive testing anxiety levels remained high (M ≥ 50.0) (diagnostic testing confirmed that the fetus had a trisomy in 10/11 women).
van Schendel, et al., 2017, Netherlands [24]	Child-related anxiety (subscale of PRAQ-R3, range 4–20)	N = 656 women with negative NIPT results	PRAQ-R3 mean score = 10.8.	PRAQ-R3 mean score = 7.8.	Women with negative NIPT result showed a significant decrease in level of child-related anxiety after receiving test results (*p* < 0.001).
Takeda, et al., 2018, Japan [23]	Psychological distress (K6, range 0–24, Japanese version)	*N* = 697 women with negative NIPT-results and low pre-NIPT psychological mental distress (K6).	Pre-NIPT K6 scores: cases (n = 29, post-partum K6 high): mean K6 score (SD, range) = 5.0 (±2.4, 0–9); controls (n = 668, post-partum K6 low: mean K6 score (SD, range) = 2.5 (±2.4, 0–9); there was no significant difference between groups.	Post-partum K6 scores: cases (*n* = 29, post-partum K6 high): mean K6 score (SD, range) = 12.8 (±3.6, 10–24); controls (*n* = 668, post-partum K6 low: mean K6 score (SD, range) = 2.0 (±2.5, 0–9); there was no significant difference between groups.	Although women may not feel mental stress before undergoing NIPT, they may develop mental distress post-partum.
Lewis, et al. 2016, UK [20]	Decisional regret (DRS, range 0–100, no formal cut-off)	N = 263 women with either negative or positive NIPT results; 1 month following receipt of results (Q2).	(−-)	DRS mean score (SD) = 3.17 (±7.27).	Very low level of decisional regret after NIPT with none of the women scoring above the midway point (0% ≥50/100).
Lo, et al., 2019, Hong Kong [21]	Decisional regret (DRS, score ≥ 50)	N = 223 women 4 weeks after baseline and after receiving results (Q2).	(−-)	DRS mean score [95% CI]: 15.7 [13.2–18.3]; DRS score ≥ 50: *N* = 13 women^a^.	Decisional regret (DRS score ≥ 50) was reported by *n* = 13 of 223 women. Among them were *n* = 200 NIPT acceptors and *n* = 23 NIPT decliners. All n = 13 women who reported decisional regret were NIPT acceptors (*n* = 12 had negative NIPT results, and n = 1 required invasive prenatal testing for either inconclusive or positive NIPT results). Decisional regret was more common in women with insufficient (n = 29) vs. sufficient (*n* = 194) knowledge about NIPT: 5/29 vs. 8/194 (*p* = 0.016).

^a^Quote from the article: “All 13 women scoring ≥50 on respiratory distress syndrome (RDS) were NIPT acceptors.” This must be a mistake, we assume that the authors refer to DRS

**Table 3:** Data from questionnaires assessing experiences with NIPT (van Schendel et al., 2017, Netherlands)

Question	Data source	Responses and/or results
Reassurance: ‘I felt reassured by the test-result’ (Scale: not at all applicable (1) – very much applicable (5))	N = 656 women with negative NIPT results.	- 2.4% not at all applicable - 0.9% hardly applicable - 15.7.% somewhat applicable - 80.9% very much applicable
Confidence: ‘I am confident that the test-result is correct’ (Scale: not at all applicable (1) – very much applicable (5))	N = 656 women with negative NIPT results.	- 0.2% not at all applicable - 0.6% hardly applicable - 18.3% somewhat applicable - 80.9% very much applicable
Certainty: ‘The test result offers me sufficient certainty whether my child has a disorder’ (Scale: not at all applicable (1) – very much applicable (5))	*N* = 656 women with negative NIPT results.	- 0.3% not at all applicable - 1.4% hardly applicable - 34.0% somewhat applicable - 64.3% very much applicable
Satisfaction with NIPT: (Scale: not at all applicable (1) – very much applicable (4)	N = 656 women with negative NIPT results.	2.4% (*n* = 16) women would rather have had invasive testing than NIPT (shorter waiting time, more accurate results)
Satisfaction with NIPT: (Scale: not at all applicable (1) – very much applicable (4)	*N* = 682 women with negative or positive NIPT results.	97.5% had no regret on NIPT; 28.6% would have preferred to receive results earlier.
Experience with test offer and procedure. (Scale: completely disagree (1) – completely agree (5), compressed to 3-point scale)	N = 682 women with negative or positive NIPT results.	96.1% of participating women have been glad to have been offered NIPT, 85.9% had had sufficient time to reflect on their choice.
Waiting time for test results. (Scale: way too long (1) – way too short (5))	N = 682 women with negative or positive NIPT results.	Reported waiting time until NIPT result: mean = 15 days (range 5–32 days). Waiting time was considered (much) too long by 68.5% of women, for 31.5% it was neither too long nor too short. A waiting time of ≤10 days was acceptable for most women, longer was considered too long by the majority of women.

**Table 4:** Quotes from interviews or focus groups after receiving NIPT results

Study	Number of participating women	Quotes of participating women	Results
Vanstone, et al., 2015, Candada [25]	N = 38 women at high risk for fetal aneuploidy; here, only quotes of women who clearly were interviewed after NIPT are considered.	Timing: “It took almost three weeks.. .. I was concerned because I knew the amnio had to happen at a certain time and that if we had to make any decisions regarding the pregnancy that had to happen at a certain time.” “I was already at 19 weeks, so I wanted to do it all fast, because if you did want to abort or anything, god forbid, they say you should do it before 22 weeks, so it was kind of like I had maybe a week or two, not even.” “You feel like it is ticking. It’s like, everything is just building your anxiety.” “I found out early enough that I’m able to have the two week wait [for NIPT results]. I’m still able to possibly have an amnio if I need it.” Accuracy: “Chance of not getting a result from the NIPT. .. to go through the test and to not actually have anything, you know time is ticking, so just making sure that we are going to get a reasonably good answer in a good amount of time was important.” “If it comes back negative, you’re pretty much fine not to worry about it because they are very accurate tests.”	Timing: The wait for results was typically described as very stressful, as the “deadline” for confirmatory testing and termination loomed. The idea of a deadline refers to the gestational age after which confirmatory invasive testing and pregnancy termination are no longer available. Women considering termination discussed perceptions of deadlines to make decisions about further testing or termination, describing the process of prenatal testing as a race against the clock. Women made frequent references to time pressures. Accuracy: The possibility of an inconclusive result was stressful for some women. Whereas women described a high confidence in negative NIPT results.
Farrell, et al., 2014, USA [19]	N = 58 women in 6 focus groups, among them *n* = 10 had NIPT; only statements of women who clearly were recorded after NIPT are considered.	Improved accuracy without risk: One woman who had NIPT commented on how she perceived the value of NIPT in her prenatal care: “In my mind, it was just as well as diagnostic. I know it’s not. I know there is still a risk but in my mind, it made me feel better that 99% is good enough for me. If there is a 1% chance of something happening, then it’s meant to happen but 99%, I could at least breathe a little easier” Identification of Fetal sex: “That is another reason why we chose to do it because we wanted to know the sex of the baby. So I knew 5 weeks before I would have known from the ultrasound and some people (asked), ‘How do you know already?’”	The values and opinions expressed about NIPT by women who have personal experience with this technology are inconsistent with the way it is has typically been implemented in Ontario so far. A revision of the current policy should consider this evidence that women value early access to accurate tests without associated risks of miscarriage when considering how and when NIPT should be implemented into the prenatal testing care pathway.

As the authors of the included studies used different expressions to describe NIPT results, we harmonized the use of the expressions throughout our results section and in the tables, as follows: ‘negative NIPT result’ includes e.g. ‘normal’ or ‘low-risk’ NIPT result, ‘positive NIPT result’ includes e.g. ‘abnormal’ or ‘high-risk’ NIPT result.

## Results

**Fig. 1:**
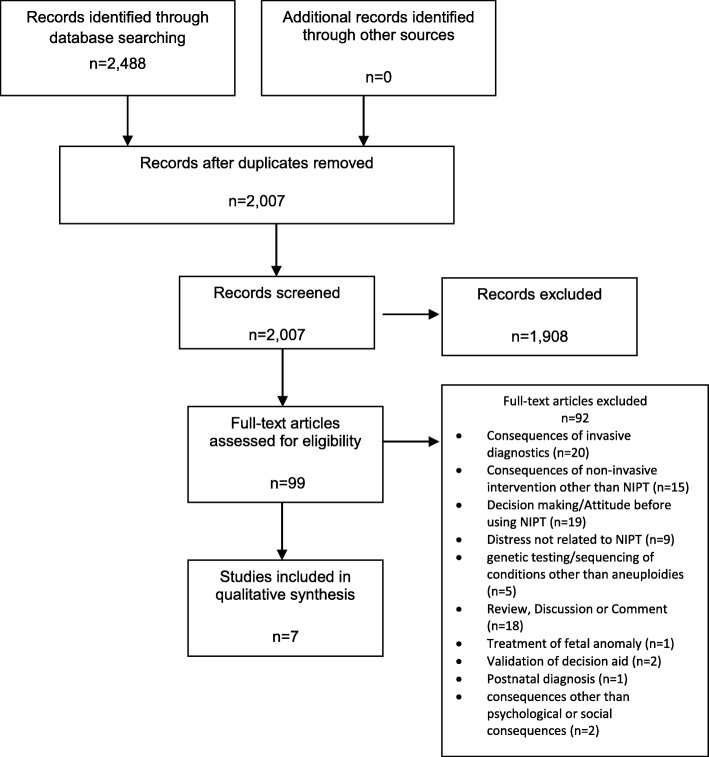
PRISMA Flow Chart

We identified a total of 2488 records. After removal of duplicates, 2007 titles and abstracts were screened; subsequently, the full-texts of 99 articles were assessed. 7 studies have been included (see PRISMA flow chart in Fig. 1).

### Characteristics of the included studies and women therein

Five studies assessed anxiety, psychological distress and/or decisional regret with validated psychological tests like the Spielberger State-Trait Anxiety Inventory (STAI or short form STAI-6), the Pregnancy-Related Anxiety Questionnaire-Revised (PRAQ-R), the Kessler Psychological Distress Scale (K6) or the Decisional Regret Scale (DRS) [20–24]. Questionnaires usually were administered at baseline (i.e. at NIPT blood draw or counseling) and after receiving NIPT results. One study conducted a survey on satisfaction and experience with NIPT [24]. Two studies assessed women’s experiences with and feelings after NIPT in interviews or focus groups [19, 25]. For more details, see Table 1.

The studies were published between 2014 and 2018 with data being assessed from 2013 until 2016 on. Two studies each were conducted in Europe [20, 24], Asia [21, 23] or North America [19, 25], one study was from Australia [22]. Four studies were single center studies [19, 22, 23, 25], three studies were multi-centric [20, 21, 24]. Five studies assessed outcomes with self-administered questionnaires [20–24], one study conducted interviews [25], and one study worked with focus groups [19].

The number of participating women in the studies assessing outcomes with questionnaires ranged from 115 to 697; in the two studies that conducted interviews or focus groups, 38 and 53 women participated [19, 25].

Five studies reported a mean age of participating women [19, 20, 22, 23]: The mean age of all included women over those five studies was 35.9 years, with the youngest and oldest age of 19 and 49 (extremities of range reported; standard deviation not reported in all studies). One study only reported the mean age of women when giving birth of 35.4 years and age classes in steps of 5 years (25–29; 30–34; 34–39; ≥40) [25], the other study reported women’s age in two groups (< 35; ≥35) [21], with the majority of women in those two studies being 35 years or older.

Most studies recruited women with an elevated Down syndrome screening (DSS) risk (medium and/or high risk, cut-offs vary, range from > 1:1000 to 1:1–125) [20–22, 24, 25], two studies recruited from an unselected population regarding DSS [19, 23].

In all studies, psychological or social consequences of women who had NIPT were assessed (e.g. anxiety, distress after NIPT or experiences with NIPT); two studies also comprised women who had not undergone NIPT [19, 21]. See below for a detailed description of the outcomes.

Takeda et al. only included women with negative NIPT results [23], in the study of Richmond et al. all women received negative NIPT results (*n* = 113 (of 115) women received a negative result, *n* = 2 women were excluded due to failed NIPT) [22]. Lewis et al., Lo et al., and van Schendel et al. included women with either negative or positive NIPT results [20, 21, 24]. Farrell et al. and Vanstone et al. did not report on NIPT results [19, 25].

All studies except one [21] reported limitations, for example, lack of control group (i.e. without NIPT), low response rates (i.e. to initial recruiting or second questionnaires), or origin of participating women from a selected community (i.e. older and highly educated).

### Description of the assessed outcomes

#### Anxiety

Four studies assessed anxiety with the Spielberger State-Trait Anxiety Inventory (STAI or short form STAI-6) [20–22, 24].

In the studies of Lewis et al., Richmond et al., and van Schendel et al. women experienced a decrease in state anxiety from baseline to the time point after receiving NIPT results (reported by a decrease in STAI mean scores). Richmond et al. (*n* = 113) and van Schendel et al. (*n* = 656) report the decrease in state anxiety for women with negative NIPT results, the women in Lewis et al.’s study (*n* = 263) received either negative or positive NIPT results.

Richmond et al. compared state anxiety in two subgroups of women: women with a high-risk combined first trimester screening (cFTS) result were significantly more anxious at baseline compared to women with a low-risk cFTS result; nevertheless, both groups experienced similar levels of state anxiety 1 week after receiving NIPT results.

The rate of women experiencing elevated anxiety (i.e. a STAI-6 score ≥ 50/80) significantly decreased from baseline to the time point after receiving NIPT results in the studies of Lewis et al. (*n* = 263) and Lo et al. (*n* = 254). Van Schendel et al. reported that the subgroup of women receiving positive NIPT results (n = 26) experienced high levels of anxiety after receiving those.

Van Schendel et al. (*n* = 656) assessed child-related anxiety with a subscale of the Pregnancy-Related Anxiety Questionnaire-Revised (PRAQ-R). Women who received negative test results experienced significantly lower levels of child-related anxiety after receiving negative test results compared to baseline. For more details, see Table 2.

#### Psychological distress

One study assessed psychological distress with the Kessler Psychological Distress Scale (K6) after NIPT in a case-control setting [23]. All women in Takeda et al.*’*s study (*n* = 697) had received negative NIPT-results and experienced low psychological distress (i.e. low K6 scores) at baseline. The women who were assigned to the case group (*n* = 29) experienced psychological distress after giving birth (i.e. high post-partum K6 scores), the women in the control group (*n* = 668) did not experience psychological distress after giving birth (i.e. low post-partum K6 scores). Factors that contributed to psychological distress in women post-partum were for example low birth weight or primiparity. For more details, see Table 2.

#### Decisional regret

Two studies assessed decisional regret (i.e. distress or remorse) after women’s decision for NIPT with the Decisional Regret Scale (DRS) [20, 21]. In the study of Lewis et al. (*n* = 263) decisional regret was very low among women after receiving (positive or negative) NIPT test results. In the study of Lo et al. (*n* = 223) decisional regret was low: among the *n* = 13 women experiencing decisional regret, *n* = 12 had received negative NIPT results. Women with insufficient knowledge about NIPT experienced decisional regret more commonly. For more details see Table 2.

#### Experiences with NIPT

Van Schendel et al. assessed experiences with NIPT in questionnaires [24]. Either a group of women with only negative NIPT results or a group of women with negative or positive results were queried. Women with negative NIPT results (*n* = 656) mostly felt sufficiently reassured by the test result and were confident that the result was correct and that their child was not affected by a disorder. Some of the women with negative NIPT results (*n* = 16) would have preferred invasive testing over NIPT because of a shorter waiting time and more accurate results.

Of the women with either negative or positive NIPT results (*n* = 682), the majority had no regret about NIPT. About a third of those women would have preferred to receive results earlier; the reported mean waiting time of 15 days (range 5–32 days) was considered too long by about two thirds of women. A waiting time of ≤10 days would have been acceptable for most women. For more details, see Table 3.

Vanstone et al. interviewed *n* = 38 women with the aim of identifying their values about publicly funded NIPT to inform future formal policy making [25]. Here, we only considered statements that had clearly been made *after* NIPT. Due to anonymization, we cannot tell from how many different women the quotes originate. Waiting time for results was an aspect that was described as very stressful, especially for women who considered either confirmatory invasive testing or pregnancy termination, both of which are only available at a certain gestational age. The possibility of an inconclusive result was also stressful for some women, whereas they described a high confidence in negative NIPT results. For more details, see Table 4.

Among the *n* = 58 women who participated in the focus groups of Farrell et al., only *n* = 10 had NIPT. Here again, we only considered statements that were clearly made after NIPT. One woman stated that the diagnostic accuracy of NIPT was sufficiently reassuring for her and that she almost considered it as confirmative. One woman reported that she had known the fetal sex of her unborn child 5 weeks earlier than would have been possible with ultrasound. This latter aspect was the only one we could find on social aspects among all the included studies. For more details, see Table 4.

## Discussion

In this scoping review, we identified seven studies that investigated psychological and/or social consequences after NIPT [19–25].

The studies that evaluated anxiety [20–22, 24] (state, elevated, or child-related) assessed outcomes either in women who had all received positive NIPT results [24], or in women who all had received negative NIPT results [22, 24], or in a mixed group of women [20]. None of the studies had a control group without NIPT, nor were comparisons made between women who had positive NIPT results and women who had negative NIPT results. Considering all studies reporting on anxiety, we cannot know if the observed decrease in anxiety is related to NIPT (or the NIPT result). A recent study (not included) on *n* = 37 women showed, that state anxiety levels (STAI) in pregnant women also decreased after ultrasound exams [26].

Similarly, in the two studies [20, 21] that found low levels of decisional regret after receiving NIPT results, the participating women had received either positive or negative results; no control group without NIPT was examined in the studies. Interestingly, among the *n* = 13 women experiencing decisional regret in the study of Lo et al. [21], all but one had received negative NIPT results.

Psychological distress was assessed among women with negative NIPT findings in one study in a case-control setting [23], wherein cases were defined as having developed psychological distress after giving birth. The authors identified several factors like parity or the mode of conception as factors for psychological distress, but again, NIPT as a factor itself could not be identified as one of those factors due to study design (no control group without NIPT).

Three studies qualitatively assessed experiences with NIPT via questionnaires, interviews, or focus groups. In the study of Van Schendel et al. [24] subgroup analysis for women with only positive NIPT findings were not feasible, although aspects like reassurance or confidence in the test results would have been interesting for both subgroups of women, i.e. for women with positive or negative NIPT results. Quotes from women from interviews (Vanstone et al. [25]) or focus groups (Farrell et al. [19]) on the aspects of waiting time and accuracy of test results correspond to Van Schendel et al.’s findings from questionnaires: Waiting time was experienced as long and/or stressful, and women had a high confidence in negative NIPT results. Waiting times for other (invasive) prenatal tests are comparable; for example, results from amniocentesis are usually available after 3 weeks.

A social aspect that only Farrell et al. [19] reported on is the possibility of the early identification of fetal sex with NIPT. This might raise issues in societies where one sex is preferred over another and an early detection might enable earlier termination of pregnancies. For this reason in Germany, for example, the identification of fetal sex will only be communicated after week 12 post-conception, when abortion without medical indication is not possible anymore [27].

We could not identify studies on the long-term psychological or social consequences of NIPT, such as decisional regret after several years. Because negative NIPT results usually do not entail further diagnostics, especially the long term psychological or social outcomes after positive NIPT results might be of interest. On an individual level, the psychological outcomes of women who receive a positive NIPT result and either chose to have or not to have confirmative invasive diagnostics could be interesting. Comparative studies on anxiety or levels of stress during pregnancy could elucidate those questions.

On population level, questions on the long term consequences of NIPT could be interesting, for example, whether the societal acceptance of children with fetal anomaly changes, or also if issues such as early abortions due to a not preferred sex of the fetus become acceptable.

Studies on psychological or social consequences of women’s partners have not been found. Carlsson et al. interviewed expectant fathers of fetuses that were diagnosed with a congenital heart defect and found that fathers also experienced intense emotional shock and are at risk of not receiving adequate support, because they set their needs aside to support their partner [28].

Considering that NIPT was introduced 8 years ago, the number of included studies at this point appears to be relatively low. In comparison to the number of studies that were included in a Cochrane Review on the diagnostic test accuracy of NIPT (*n* = 65 from 2007 to 2016) [1], the available data on psychological or social consequences of NIPT is considerably more scarce. Our findings support a statement of Bryant from 2014: *“the effect of test characteristics and the social context in which they are offered has been one of the least researched topics in the psychology of screening”* [29].

NIPT being a non-invasive test might remove the parent’s fear of harming the fetus in an invasive procedure; however, the anxiety and distress that are often related to a diagnostic situation remain. Çakar et al., identified the fear of receiving bad news as a main factor why patients feel anxious before an invasive procedure. Moreover, they found that patients who had received information from doctors or nurses had lower anxiety levels compared to patients who had either received no information or information from friends and family [30]. The fact that NIPT is only available in a controlled medical environment (and not for sale on the internet) might help to assure good quality counseling and reduce patients’ anxiety. Distress in non-invasive prenatal screening situations other than NIPT was found to be reduced by delivering information about testing correctly and thus helping patients to make informed decisions [31].

Good counseling is even more important for NIPT because parents might only be aware of the benefits of NIPT and might not anticipate the consequences. Because the positive predictive value of a test correlates with the prevalence of the disease in the population tested, the test accuracy is better in populations with a high risk [32]. If NIPT was offered routinely in obstetrical care (i.e. for women that are not at high risk of fetal aneuploidy), the rate of false positive results would increase. Hence, more women, who did not consider themselves to be at risk, would be confronted with false positive results and possibly experience psychological distress.

NIPT is being expanded beyond trisomy 21 and chromosome aneuploidies; screening for the fetal rhesus D status or monogenic diseases like thalassemia is already possible [33]. The future development of NIPT for a panel of relatively rare genetic disorders (i.e. that have a low prevalence in the general population) would similarly result in a higher rate of false positive results for women who are not at a high risk. Consequences might thus be further testing and increased anxiety, especially if knowledge about certain diseases and treatment options is little. Moreover, parents might not be aware of the fact that NIPT can only assess a small subset of anomalies and that a negative NIPT result does not give full certainty about the health of the fetus.

In our scoping review, we included a limited number of studies that assessed psychological and social outcomes with different methodologies and with women from different countries and cultural contexts. We assume that those results would also apply to populations comparable to the ones studied, and would thus also be applicable to Germany or other European countries.

## Conclusions

More research on the psychological and social consequences is desirable, since NIPT might become a part of prenatal care in many countries. In 2019, for instance, the German Federal Joint Committee (Gemeinsamer Bundesausschuss, G-BA) will decide on the reimbursement of NIPT by statutory health insurance funds (Gesetzliche Krankenversicherung, GKV) in pregnancies with a high risk of anomalies. Of note, it is not planned to be used as a routine screening test.

It is important to make expecting parents aware of possible short- and long-term psychological and social consequences. The mapping of studies that analyze psychological and social consequences of NIPT is one step toward identifying research gaps and encouraging new research so that there will be a sound empirical basis that both physicians and parents can use to make fully informed decisions on the use of NIPT.

## Supplementary information

**Additional file 1.** Search strategies for the databases searched.

## Data Availability

The dataset used and analyzed during the current study is available from the corresponding author on reasonable request.

## Abbreviations

AMA: Advanced maternal age
cffDNA: Cell-free fetal DNA
cFTS: Combined first trimester screening
CI: Confidence interval
COI: Conflicts of interest
DRS: Decisional Regret Scale
DSS: Down syndrome screening
FCT: First-trimester combined test
K6: Kessler psychological distress scale
n: Number
NIPT: Non-invasive prenatal testing
ORCID: Open researcher and contributor ID
p: *p*-value
PRAQ-R: Pregnancy-related anxiety questionnaire-revised
Q1, Q2: Questionnaire 1, questionnaire 2
SD: Standard deviation
STAI: Spielberger State-Trait Anxiety Inventory
vs: Versus
ys: Years
