# Supplementary material for: Psychological and social consequences of non-invasive prenatal testing (NIPT): a scoping review
Source: BMC Pregnancy Childbirth. 2019 Oct 28;19:385. doi: 10.1186/s12884-019-2518-x (PMC6819451; doi:10.1186/s12884-019-2518-x)
Supplement: Supplementary file 1 — Additional file 1. Search strategies for the databases searched. [file 12884_2019_2518_MOESM1_ESM.pdf]

Appendix 1: Search strategies

Medline (Ovid)

Database(s): Ovid MEDLINE(R) 1946 to July Week 4 2018, Ovid MEDLINE(R) Daily Update August 07, 2018, Ovid MEDLINE(R) In-Process & Other Non-Indexed Citations August 07, 2018, Ovid MEDLINE(R) Epub Ahead of Print August 07, 2018

ID	Query	Results
1	MOTHERS/	37574
2	FATHERS/	7993
3	Pregnant Women/	6829
4	pregnan*.ti,ab,kf.	476435
5	mother*.ti,ab,kf.	196883
6	matern*.ti,ab,kf.	249847
7	father*.ti,ab,kf.	37635
8	parent*.ti,ab,kf.	376695
9	wom?n.ti,ab,kf.	1031570
10	m?n.ti,ab,kf.	1391484
11	couple*.ti,ab,kf.	319617
12	or/1-11	3200741
13	Prenatal Diagnosis/	34959
14	Genetic Testing/	33358
15	((prenatal or aneuploid*) adj3 (screen* or diagnos* or test*)).ti,ab,kf.	31245
16	((non-invasive or noninvasive) adj5 (prenatal test* or prenatal diagnos* or genom* based)).ti,ab,kf.	1577
17	(gNIPT or NIPT).ti,ab,kf.	534
18	or/13-17	82254
19	exp Maternal Behavior/	11484
20	exp Adaptation, Psychological/	118205
21	Anxiety/	70893
22	Social Support/	64160
23	Decision Making/	84443
24	(Anxiety or anxious).ti,ab,kf.	168977
25	Attitude*.ti,ab,kf.	132672
26	Psycho*.ti,ab,kf.	600175
27	Crisis*.ti,ab,kf.	43844
28	Distress*.ti,ab,kf.	107439
29	Trauma*.ti,ab,kf.	326230
30	Stress.ti,ab,kf.	644084
31	Depression.ti,ab,kf.	296585
32	or/19-31	2123581

33	12 and 18 and 32	4214
34	limit 33 to yr="2011 -Current"	1331

Cochrane Library

Date run: 08/08/2018 08:42:19

ID	Query	Results
#1	MeSH descriptor: [Mothers] this term only	1482
#2	MeSH descriptor: [Fathers] this term only	152
#3	MeSH descriptor: [Pregnant Women] this term only	176
#4	pregnan* or mother* or matern* or father* or parent* or wom?n or m?n or couple*	276821
#5	#1 or #2 or #3 or #4	276821
#6	MeSH descriptor: [Prenatal Diagnosis] this term only	169
#7	MeSH descriptor: [Genetic Testing] this term only	361
#8	(prenatal or aneuploid*) near/3 (screen* or diagnos* or test*)	943
#9	(non-invasive or noninvasive) near/5 ((prenatal test*) or (prenatal diagnos*) or (genom* based))	1279
#10	gNIPT or NIPT	20
#11	#6 or #7 or #8 or #9 or #10	2507
#12	MeSH descriptor: [Maternal Behavior] explode all trees	276
#13	MeSH descriptor: [Adaptation, Psychological] explode all trees	4925
#14	MeSH descriptor: [Anxiety] this term only	6457
#15	MeSH descriptor: [Social Support] this term only	3016
#16	MeSH descriptor: [Decision Making] this term only	1977
#17	anxiety or anxious or attitude* or psycho* or crisis* or distress* or trauma* or stress* or depression	227826
#18	#12 or #13 or #14 or #15 or #16 or #17	229023
#19	#5 and #11 and #18 with Cochrane Library publication date between Jan 2011 and Aug 2018	248

CINAHL

Date run: August 08, 2018 5:54:43 AM
Search modes: Boolean/Phrase

ID	Query	Results
S7	S1 AND S4 AND S5 (Limits: Published date: 20110101-20181231)	400
S6	S1 AND S4 AND S5	1,135
S5	anxiety or anxious or attitude or psycho* or crisis* or distress*	711,392

	or trauma* or stress or depression	
S4	S2 or S3	5,886
S3	("non-invasive" or "noninvasive") N5 ("prenatal test*" or "prenatal diagnos*" or "genom* based")	235
S2	(prenatal or aneuploid*) N3 (screen* or diagnos* or test*)	5,886
S1	pregnan* or mother* or matern* or father* or parent* or wom?n or m?n or couple*	493,384

PsychINFO

Date run: August 08, 2018 6:06:14 AM
Search modes: Boolean/Phrase

ID	Query	Results
S7	S1 AND S4 AND S5 (Limits: Publication year 2011-2018)	509
S6	S1 AND S4 AND S5	1,393
S5	anxiety or anxious or attitude or psycho* or crisis* or distress* or trauma* or stress or depression	3,280,241
S4	S2 or S3	2,383
S3	("non-invasive" or "noninvasive") N5 ("prenatal test*" or "prenatal diagnos*" or "genom* based")	46
S2	(prenatal or aneuploid*) N3 (screen* or diagnos* or test*)	2,383
S1	pregnan* or mother* or matern* or father* or parent* or wom?n or m?n or couple*	824,877
